# Low-Dose Computed Tomography for the Optimization of Radiation Dose Exposure in Patients with Crohn's Disease

**DOI:** 10.1155/2018/1768716

**Published:** 2018-10-31

**Authors:** Richard G. Kavanagh, John O'Grady, Brian W. Carey, Patrick D. McLaughlin, Siobhan B. O'Neill, Michael M. Maher, Owen J. O'Connor

**Affiliations:** ^1^Department of Radiology, Cork University Hospital, Cork, Ireland; ^2^Department of Radiology, University College Cork, Cork, Ireland; ^3^Department of Gastroenterology, Cork University Hospital, Cork, Ireland; ^4^APC Microbiome Ireland, University College Cork, Cork, Ireland

## Abstract

Magnetic resonance imaging (MRI) is the mainstay method for the radiological imaging of the small bowel in patients with inflammatory bowel disease without the use of ionizing radiation. There are circumstances where imaging using ionizing radiation is required, particularly in the acute setting. This usually takes the form of computed tomography (CT). There has been a significant increase in the utilization of computed tomography (CT) for patients with Crohn's disease as patients are frequently diagnosed at a relatively young age and require repeated imaging. Between seven and eleven percent of patients with IBD are exposed to high cumulative effective radiation doses (CEDs) (>35–75 mSv), mostly patients with Crohn's disease (Newnham E 2007, Levi Z 2009, Hou JK 2014, Estay C 2015). This is primarily due to the more widespread and repeated use of CT, which accounts for 77% of radiation dose exposure amongst patients with Crohn's disease (Desmond et al., 2008). Reports of the projected cancer risks from the increasing CT use (Berrington et al., 2007) have led to increased patient awareness regarding the potential health risks from ionizing radiation (Coakley et al., 2011). Our responsibilities as physicians caring for these patients include education regarding radiation risk and, when an investigation that utilizes ionizing radiation is required, to keep radiation doses as low as reasonably achievable: the “ALARA” principle. Recent advances in CT technology have facilitated substantial radiation dose reductions in many clinical settings, and several studies have demonstrated significantly decreased radiation doses in Crohn's disease patients while maintaining diagnostic image quality. However, there is a balance to be struck between reducing radiation exposure and maintaining satisfactory image quality; if radiation dose is reduced excessively, the resulting CT images can be of poor quality and may be nondiagnostic. In this paper, we summarize the available evidence related to imaging of Crohn's disease, radiation exposure, and risk, and we report recent advances in low-dose CT technology that have particular relevance.

## 1. Introduction

Crohn's disease is characterized by transmural inflammation that may affect any part of the gastrointestinal tract, and it is a life-long condition that relapses and remits throughout its course [1]. Improved understanding of the pathogenesis of Crohn's disease combined with recent availability of immunomodulatory treatments has expanded the range of medical therapies available to physicians who treat patients with Crohn's disease [2]. Tailored imaging investigations are a key component of the decision-making process for a number of reasons. First, determining the extent and activity of Crohn's disease informs treatment-related decisions. This requires radiological evaluation of small intestine and extra intestinal manifestations as well as endoscopic (gastroscopy and colonoscopy) and laboratory investigations [3]. Second, monitoring disease progression and response to treatment including surgery using imaging allows therapeutic optimization. Appropriate investigations allow early detection of complications, which potentially require surgical treatment including fibrostenotic disease, which can cause bowel obstruction, or fistulating disease which can lead to abscess formation ([4]). Third, treatment side effects range from nausea, which may limit compliance, to an increased risk of lymphoproliferative disorders, lymphoma, melanoma, and nonmelanoma skin cancers associated with immunosuppressant [5–7]. Similarly, chronic inflammation of the gastrointestinal tract increases the risk of colorectal cancer [5–7]. These associations sometimes necessitate surveillance strategies for patients with Crohn's disease [4].

## 2. Nonionising Radiation Modalities

The use of imaging modalities that do not require ionizing radiation is the preferred method of reducing radiation exposure among patients with Crohn's disease. These include magnetic resonance imaging (MRI), ultrasound, and capsule endoscopy.

### 2.1. MRI

MRI is used to a great effect to identify both acute and chronic features of Crohn's disease [8]. Magnetic resonance enterography (MRE) is the preferred method of small bowel cross-sectional imaging. This is partly due to concern regarding cumulative ionizing radiation exposure from CT and fluoroscopy, especially in children and young adults, who will undergo many examinations throughout their life, otherwise amassing a potentially significant cumulative radiation exposure [9]. MRI is well suited for evaluating small-bowel inflammatory disease with reported sensitivity of 93% and specificity of 93% [10]. MRI offers superior soft tissue contrast resolution, multiplanar capability, and the potential of obtaining functional information. The main indications for MRE include small bowel imaging in patients with suspected or surveillance of known Crohn's disease. The examination may also be combined with the assessment of perianal disease, which is also optimally performed using MRI.

The absence of ionizing radiation is a strong advantage of MR imaging. These advantages often outweigh the disadvantage of the relatively long time it takes to perform MR enterography and increased cost relative to CT [11, 12]. In the clinical setting of a critically ill patient, the MRI suite presents many additional challenges over CT in terms of patient safety, especially regarding monitoring lines and support equipment which need to be nonferromagnetic. MRI imaging in the setting of Crohn's disease also often requires the administration of a gadolinium-based contrast agent (GBCA), which has recently come under increasing scrutiny due to gadolinium deposition in the dentate nuclei, pons, globus pallidus, and thalamus of patients undergoing multiple MRIs requiring GBCA administration [13, 14]. The clinical significance of this deposition is as yet unknown but has led to the European Medicine Agency's Pharmacovigilance Risk Assessment Committee recently recommending the suspension of marketing authorization for four linear GBCAs [15].

### 2.2. Diffusion-Weighted Imaging

Diffusion-weighted imaging (DWI) can be used as a valuable sequence for the depiction of lesions and can change the MRI protocol and obviate the need for gadolinium administration. While long used in other parts of the body such as the brain, the use of DWI to assess bowel is relatively new. Increased T2 signal intensity and restricted diffusion on DWI of the bowel wall have been shown to relate to acute inflammation [16].

The ability of DWI to differentiate between actively inflamed small bowel segments and normal small bowel in CD has been demonstrated, showing superior sensitivity versus dynamic contrast-enhanced MR [17]. A prospective study involving 31 patients with CD compared DWI with conventional MRE in estimating small bowel inflammation. DWI hyperintensity was highly correlated with disease activity evaluated using conventional MRE [18]. DWI has also been shown to complement T2-weighted imaging of the internal fistula and sinus tracts [19].

However, improved spatial resolution to facilitate thinner image slices is required before DWI can replace gadolinium-enhanced sequences or be used as a reliable quantitative biomarker for monitoring disease activity [20].

Recent developments in MRI technology, such as faster gradient sequences and refined receiver coils, will boost its convenience and allow for more efficient MR imaging. Not only should these advances increase patient through-put but these will also reduce motion artifact and improve image spatial resolution which are current limitations of MRI compared with CT. Being radiation-free, these advances are most significant for younger cohorts of patients with CD and those undergoing serial and repeated imaging studies for known CD [21].

### 2.3. Ultrasound

The pathognomonic finding of Crohn's disease is discontinuous and dishomogeneous transmural inflammation extending through all layers of the intestinal wall. The presence of these features forms the basis of ultrasound (US) diagnosis of Crohn's disease. Standard B-mode ultrasound is of limited utility in this setting, but some recent papers have suggested that contrast-enhanced US may be of use in determining disease activity [22–24], although this is slow to be implemented into widespread clinical practice.

US assessment in patients with CD typically reveals stiff and thickened bowel walls variably associated with an alteration of normal peristaltic activity in the small bowel as well as the absence of colonic haustral folds. Contrast-enhanced, power, and color Doppler ultrasound allow for increased accuracy in the assessment of the small bowel CD [25]. Recent studies have shown that US reliably locates and characterizes inflammatory infiltration of the bowel wall and assesses local abnormalities such as abscess formation [26]. A significant problem with the use of ultrasound in this setting, however, is that it is heavily operator-dependent and is extremely time-consuming.

### 2.4. Capsule Endoscopy

Capsule endoscopy (CE), introduced in 2000 [27], is an increasingly available method for assessing small intestinal pathology. Current indications for CE include the identification of obscure gastrointestinal tract bleeding (OGIB) and investigation of Crohn's disease, small intestine tumours, and malabsorptive states [28]. In the evaluation of Crohn's disease, stricturing or penetrating disease increases the risk of capsule retention (defined as the capsule remaining in the gastrointestinal tract for longer than 2 weeks [28]) and capsule perforation [29, 30]. CT and MRI techniques to determine luminal patency are useful prior to CE. Importantly, MRI use is contraindicated in cases of capsule retention [28], which may occur due to gastroparesis and motility disorders, as well as for mechanical reasons secondary to complications of Crohn's disease [28, 30]. Furthermore, nonspecific mucosal abnormalities are often detected with CE and, without biopsy capability, this can lead to high false positive rates of Crohn's disease [29], reducing benefits of CE over radiological modalities. Capsule aspiration is a rare complication, most often seen in patients with neurological or swallowing disorders and reduced or absent cough [29]. In carefully selected cases, particularly stable OGIB and nonstricturing Crohn's disease, CE is a safe, noninvasive investigative tool that reduces radiation exposure for patients in the evaluation of small intestinal mucosa.

## 3. Computed Tomography

### 3.1. Background

CT uses ionizing radiation in the form of X-rays, to form an image of a patient. The traditional method of image reconstruction (i.e., the reconstruction algorithm) used by the computer to form the images is called filtered back projection (FBP). This method relies on the patient being exposed to a relatively large dose of radiation in order to create diagnostic quality images. Image noise becomes an issue at low radiation doses with FBP, and images with large amounts of noise can significantly impair the ability of the interpreting radiologist to form an accurate opinion of the images. Recent advances in the computational power and efficiency have facilitated the use of iterative reconstruction of CT images. These new iterative reconstruction (IR) techniques have some major advantages over FBP: they reduce image noise, reduce the occurrence of artefacts (e.g. streak from metallic implants), and facilitate the acquisition of CT images at much lower radiation doses while maintaining diagnostic image quality [31].

### 3.2. Radiation Dose

Three main metrics are used to estimate patient radiation exposure in CT. The radiation dose output from the scanner is represented by the CT dose index (CTDI)_vol_ measured in milligrays (mGy); the dose over the total length of the scan is represented by the dose-length product (DLP) measured in mGy·cm; the effective dose (ED), measured in millisieverts (mSv), which represents the equivalent whole-body dose that would have the same risk of the biologic effect, can be derived by multiplying DLP by a conversion factor based on the CT scan parameters and the body part imaged [32]. A standard CT of the abdomen and pelvis (CT-AP) exposes the patient to an ED of approximately 8 mSv although values reported in the literature range from 3.5 mSv to 25 mSv [33]. The radiation exposure associated with CT-AP is significantly more than the annual natural background radiation of 3–4 mSv received by the average person from the environment [34].

### 3.3. Risks of Radiation Exposure

High-dose radiation exposure leads to predictable deterministic effects that only occur above a certain threshold dose, and the severity of the injury once this threshold is reached is dose dependent; examples of this include skin burns (threshold 2 Gy) [35, 36] and cataract formation (threshold 0.5 Gy) [37]. However, even below these thresholds, exposure to low-level ionizing radiation is associated with stochastic effects; these are probabilistic effects that are unrelated to dose and are responsible for cancer induction in human cells.

The current widely accepted model of cancer risk from low-level radiation exposure is called the linear no-threshold model, whereby any exposure to ionizing radiation, however small, has the potential to cause harm. Several clinical studies have attempted to quantify cancer risk from CT radiation exposure [38, 39], but this is very difficult to accurately perform as the increased risk due to diagnostic imaging is small and it is extremely difficult to control for confounding factors in the required large study population over a long time period. Another difficulty in risk quantification is that there is usually a latent time from radiation exposure to cancer development of many years.

Age at the time of radiation exposure is known to be an independent risk factor for subsequent cancer mortality [40], and this is particularly relevant in the setting of Crohn's disease where most patients are diagnosed between the ages of 15 and 40; one large US epidemiological study reported a median age at the diagnosis of 29.5 years [41].

Patient knowledge of the risk associated with radiation exposure is generally low [42], and the information available to these patients, primarily from the internet, can be of questionable accuracy [43], so it is the responsibility of referring physicians and radiologists to communicate these risks to patients in an easily understandable and effective way. Guidance on how to source accurate information on the internet may be very helpful to patients, similar to the way in which physicians appraise medical literature. This includes factors such as the presence of Health on the Net Foundation Code of Conduct Certification (HONcode), an identifiable author, and references to the peer-reviewed literature [43].

Some published estimates of cancer risk include a 3-fold increased risk of leukaemia with 50 mGy exposure as a child [38], a 3-fold risk of brain cancer with 60 mGy exposure as a child [38], the induction of 125 breast cancers per 100,000 women screened between ages 40 and 74 [44], and a 1.8% increase in lung cancers if 50% of the population between aged 50 and 75 were screened for lung cancer with CT annually [45].

However, the issue of estimation of risk associated with exposure to ionizing radiation in the diagnostic range remains extremely controversial. With increased attention to this subject in the media and more alarmingly on the internet and in social media, physicians must ensure that misinformation does not lead to situations where clinically indicated CT scans are being refused by patients because of exaggerated fears of developing malignancy.

Medical imaging now accounts for approximately 50% of total population radiation dose [46], and CT accounts for approximately 60% of the dose received from medical imaging [47]. The importance of patient radiation exposure from serial CT examinations has been highlighted by the International Commission on Radiological Protection (ICRP) in its Publication 102 [48]. It is believed that any dose of radiation, however small, has the potential to cause harm, and so the increased radiation dose from CT is of concern to many [40, 49]. For now and until there is irrefutable contrary evidence, the “as low as reasonably achievable” (ALARA) principle guides radiation protection practices [50, 51].

### 3.4. CT in Crohn's Disease

CT imaging as an alternative of MRI and ultrasound is often used for imaging in Crohn's disease due to its availability, accessibility, familiarity, rapid acquisition time, and ability to evaluate mural, extramural, and extraintestinal manifestations in a single examination [3]. There are clinical circumstances where CT is the preferred method of imaging assessment, for example, in the acute setting, postoperatively, in patients with contraindications to MRI, or in claustrophobic patients. The development of low-dose CT scans can reduce patient radiation exposure which is particularly important when required for young patients with CD. It is of particular use in acutely unwell patients for the assessment of abscess formation or perforation [52]. CT enterography (CTE) is a variation of routine CT that specifically assesses the extent and severity of CD in the small bowel. It is performed with the combination of 1 litre of a neutral or low-density oral drink/beverage with intravenous iodinated contrast media. This combination optimizes luminal distention and contrast resolution in the small bowel and improves visualization of mural abnormalities such as strictures or fistulae. Diagnostic criteria for Crohn's disease using CTE include bowel wall thickening, bowel hyperemia, submucosal fat deposition, and lymphadenopathy. This cross-sectional imaging technique can also detect complications of CD including bowel obstruction, fistula, perforation, or abscess [53].

CTE is indicated in symptomatic patients, older patients (over 35 years old), and when there are contraindications to MR imaging [54]. Conventional CT is preferred for acutely unwell patients especially where there are signs of abscess formation or hollow viscus perforation.

Low-dose CTE using iterative reconstruction techniques (e.g., model-based iterative reconstruction, adaptive statistical iterative reconstruction, and sinogram-affirmed iterative reconstruction) has been found to be sensitive and specific for the detection of active inflammatory changes of CD while utilizing radiation doses significantly lower than those associated with conventional techniques. [55] There are known risk factors in Crohn's disease patients that tend to result in higher lifetime cumulative effective doses, including a history of surgery, biologic therapy, pain-predominant symptoms, isolated ileal disease, and structuring or penetrating Crohn's disease [56–58]. Between 7 and 11% of patients with IBD are exposed to high CED (>35–75 mSv), mostly patients with CD [59–62].

### 3.5. Low-Dose CT

While there is no standard definition for what constitutes a low-dose abdominal CT protocol, we consider scans where the effective dose delivered approaches that of a standard abdominal plain film or KUB radiograph to be a low-dose CT examination. There have been huge strides made recently in the attempts to reduce the radiation exposure to patients from CT. Dose reduction techniques include automatic tube current modulation [63], truncated protocols with fewer images [64], increasing acceptable image noise [65], reduced mA and kV scanning, and clinical use of new iterative reconstruction techniques [66, 67]. There is a fine balance to be struck between reducing individual patient radiation exposure and maintaining sufficient image quality to allow an accurate diagnosis to be made, and this is an area of intensive research.

An example of parameters for a low-dose abdominal CT protocol at our institution is shown in Table 1. The low-dose protocol is designed to impart a radiation exposure of 10–20% of a routine abdominal CT. The data are reconstructed using a pure iterative reconstruction algorithm (model-based iterative reconstruction, MBIR, Veo, GE Healthcare, GE Medical Systems, Milwaukee, WI). The mean effective radiation dose imparted by such a protocol is 0.83 mSv for normal weight patients increasing to 2.0 for overweight patients [67]. The typical conventional protocol effective radiation dose is 6.1 mSv. Both low-dose CT of the abdomen and pelvis and CTE can be performed using these parameters and appropriate patient preparation. It is important to highlight that imaging parameters need to be tailored towards the technology being used, the patient size, and the familiarity of the reporting radiologist with the altered appearance of a low-dose CT examination.

Low-dose CT lends itself well to thoracic imaging, partly due to the high inherent tissue contrast in the lungs. Low-dose CT in the abdomen and pelvis is challenging due to similar densities of adjacent structures and little difference between the attenuations of normal and pathological processes that can be easily obscured by increased image noise in the low-dose setting. For example, the identification of subtle stranding of the fat or prominence of the vasa recta associated with inflamed loops of small bowel is sometimes vital in the detection of active disease. Image noise in low-dose CT images may potentially impact detection of these subtleties.

**Figure 1:**
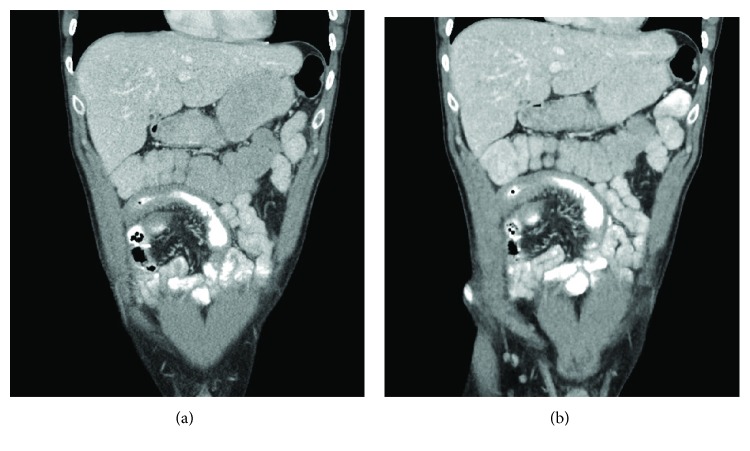
(a) Conventional dose CT reconstructed with ASIR and (b) low-dose CT reconstructed with MBIR demonstrating terminal ileum mural thickening and prominent vasa recta suggestive of acute inflammation. The low-dose CT entails an approximately 75% radiation dose reduction (reduced from 3.5 mSv to 0.98 mSv).

**Figure 2:**
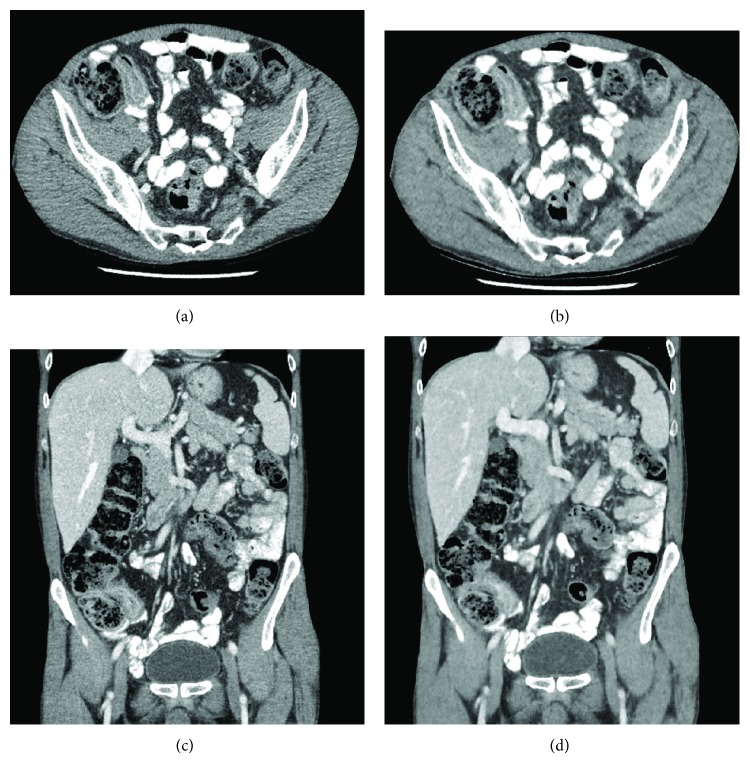
(a, c) Conventional dose axial and coronal CT reconstructed with ASIR and (b, d) low-dose axial and coronal CT reconstructed with MBIR demonstrating mural thickening at the terminal ileum and adjacent mild fat stranding consistent with acute inflammation. The low-dose CT entails an approximately 75% radiation dose reduction.

**Figure 3:**
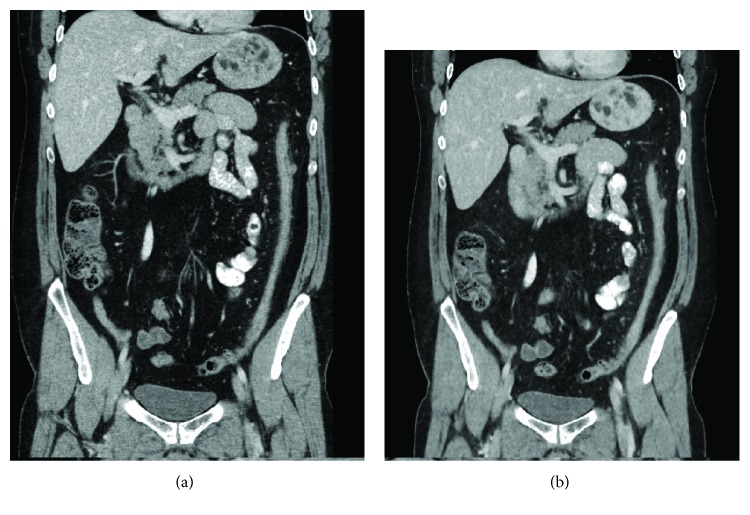
(a) Conventional dose coronal CT image reconstructed with ASIR and (b) low-dose coronal CT image reconstructed with MBIR demonstrating a thickened, featureless descending colon suggestive of chronic inflammation. The low-dose CT entails an approximately 75% radiation dose reduction.

New reconstructive algorithms, termed iterative reconstruction, use a more complex process of image formation from raw projectional data by taking into account the scanner geometry and noise statistics and in some cases mathematical models to incorporate the shape and nonlinear polychromatic nature of the X-ray beam, the focal spot geometry, and the three-dimensional shape of the voxels. This more computationally intense method of image reconstruction results in lower levels of image noise, and therefore, the CT scans may be acquired using a reduced amount of radiation while maintaining equivalent image quality. Hybrid IR methods blend FBP with a percentage of iterative reconstruction whereas pure model-based IR is a fully IR-based image reconstruction algorithm. Many IR algorithms have been shown to be reliable for image reconstruction in a number of clinical settings including but not limited to cystic fibrosis [68], urolithiasis [66], CT enterography [67], follow-up of testicular cancer [69], and carotid angiography [70] with most reporting dramatic dose reductions while maintaining diagnostic image quality. In the setting of Crohn's disease, there have been several studies detailing markedly reduced radiation exposure from CT due to the utilization of iterative reconstruction algorithms [55, 71–74], with dose reductions reported from 34 to 74% compared with standard dose CT-AP. This represents an effective dose reduction from 3.5 mSv to 0.98 mSv with no significant differences in terms of diagnostic ability reported (Figures 1–3).

Iterative reconstruction methods for low-dose CT rely on the modelling of statistical characteristics in the image domain. The current methods for this direct processing of reconstructed images lead to significant amounts of image noise. Innovative application of deep learning technology has demonstrated a great potential of deep learning for noise suppression, structural preservation, and lesion detection at a high computational speed for low-dose CT imaging [75]. This development of a specialized neural network may lend itself to future applications such as 3D and dynamic reconstruction as well as adaptation to other imaging modalities.

A recent retrospective study comparing a novel IR algorithm improved sinogram-affirmed iterative reconstruction (SAFIRE^∗^, Siemens Healthcare, Erlangen, Germany), with the standard filtered back projection (FBP). It was found that half-dose CT datasets reconstructed with SAFIRE^∗^ maintained acceptable image quality compared with full-dose CT datasets reconstructed with FBP [76]. The IR algorithm in this case, though a research-only prototype currently, potentially allows dose reductions in the order of 50% over conventional CT scans.

While preliminary low-dose protocol optimization can be performed with anthropomorphic and quality insurance phantoms, individual patient size characteristics, such as body weight, body-mass-index, and effective diameter, can readily change the performance of some dose-reduction tools. As iterative reconstruction algorithms are nonlinear, results from phantom measurements will not predict performance in humans. Also, the alteration of low-dose protocol parameters is not usually feasible during routine clinical practice because adequate diagnostic image quality must be ensured for all CT studies. This problem has led to the development of simulation tools that subsequently simulate low-dose data from clinically acquired high-dose scans allowing clinicians to improve low-dose protocols using patient data acquired during clinical practice [77].

This tool has been used to determine the lowest achievable radiation dose using iterative reconstruction for CT imaging of the appendix in young adults [78]. A 1.0 mSv appendiceal CT was found to be noninferior to 2.0 mSv CT in terms of diagnostic performance. These results point to the possibilities that simulation tools offer to clinical departments.

The projection data from previously acquired patient scans enable researchers to optimize the performance of iterative reconstruction algorithms. With standardized data sets, researchers from any discipline could evaluate IR algorithms against the results of competing methods, helping them to more rapidly determine which methods optimally reduce radiation dose [79].

With the number of CT study acquisitions increasing annually, it is paramount to further develop innovative dose reduction tools while expanding the utility of abdominal CT diagnostics.

Another important aspect of low-dose CT acquisitions is patient positioning. A recent study investigated the effect of patient off-centering in human cadavers [80]. Failure to ensure correct patient positioning resulted in a dose overestimation of up to 92%. Techniques such as laser-guided automatic patient-centering software have the potential to offer dose saving of up to 30% for chest CT and up to 56% for abdominal CT [81]. This emphasizes the valuable function such techniques have in CT organ dose conservation.

## 4. Conclusion

Patients with Crohn's disease are susceptible to high cumulative radiation exposures, particularly patients with recurrent disease and those who require steroid administration or surgery. In order to minimise radiation exposure to patients with Crohn's disease, imaging methods which do not entail ionizing radiation need to be used where possible. Nevertheless, there is a continuous global trend towards increased use of CT in medical imaging which is particularly relevant for patients with Crohn's disease who are frequently young at diagnosis and require lifelong imaging [56]. Recent developments in CT technology have the potential to considerably reduce the ionizing radiation exposure to patients with CD. Concerns remain regarding the risk of patient exposure to ionizing radiation, and with CT contributing most to medical radiation dose, it is imperative that we continue to strive for continuous improvements in patient radiation protection in order to keep radiation exposure as low as reasonably achievable. Many recent research studies have focused on the utility of new iterative image reconstruction algorithms in this regard and have highlighted the ability of these new software developments to facilitate the CT scanning at low-doses while maintaining diagnostic image quality. Future research will focus on optimizing these algorithms even further in order to achieve the minimum CT radiation dose without compromising diagnostic ability. There is little doubt that CT will retain a central role in imaging of Crohn's disease patients, but optimization of radiation exposure must remain central to future developments.

## Figures and Tables

**Table 1:** Example low-dose CT abdomen protocol.

Items	Parameters
kVp	100 kV
ATCM	20–350 mA
Rotation time	0.5 s
Noise index	85 HU
Slice thickness	64 × 0.625 mm
Reconstruction width	3 mm

ATCM = *z*-axis automated tube current modulation.
